# Calcium Signaling in Aging and Neurodegenerative Diseases 2019

**DOI:** 10.3390/ijms21031125

**Published:** 2020-02-07

**Authors:** Luísa Cortes, João Malva, Ana Cristina Rego, Cláudia F. Pereira

**Affiliations:** 1 Center for Neuroscience and Cell Biology (CNC), University of Coimbra, Rua Larga, Faculty of Medicine, Polo I, 1st floor, 3004-504 Coimbra, Portugal; lcortes@cnc.uc.pt (L.C.); a.cristina.rego@gmail.com (A.C.R.); 2 CIBB-Center for Innovative Biomedicine and Biotechnology, University of Coimbra, Rua Larga, Faculty of Medicine, Polo I, 1st floor, 3004-504 Coimbra, Portugal; jomalva@fmed.uc.pt; 3 Faculty of Medicine, Azinhaga de Santa Comba, Celas, 3000-548 Coimbra, Portugal; 4 iCRB- Coimbra Institute for Clinical and Biomedical Research; Azinhaga de Santa Comba, Celas, 3000-548 Coimbra, Portugal

**Keywords:** calcium, synapses, mitochondria, endoplasmic reticulum, glia, stem cells, calcium imaging

## Abstract

The European Calcium Society (ECS) workshop, which is held every 2 years, is a dedicated meeting of scientists interested in the elucidation of the action of calcium binding, calcium signaling and the study of proteins and organelles, such as mitochondria and endoplasmic reticulum, thereby involved, either in health and disease conditions. The 8th edition of the ECS workshop was organized by a group of researchers from the University of Coimbra, Portugal, in close collaboration with ECS board members. Thanks to the central role of “Calcium Signaling in Aging and Neurodegenerative Disorders”, the ECS 2019 workshop was attended by 62 experts who presented their results in a plenary lecture and five regular symposia, two oral communication sessions and two poster sessions, followed by a hands-on session on calcium imaging. All the scientific and social events were fully participated by the scientific community that allowed a close and fruitful interaction and discussion between junior researchers and senior experts in the field. In this report, the contributions in individual sessions are summarized.

## 1. Introduction

The European Calcium Society (ECS) aims to develop and sustain relationships between all scientists working in the field of calcium binding, calcium signaling and the study of proteins and organelles, such as mitochondria and endoplasmic reticulum (ER), as well as nuclear calcium, involved in health and disease. In recent years, the ECS could boast of a membership varying between 250 and 300 members. The members presently belong to 32 different countries (19 European countries and 13 countries from North, Central and South America, Asia and Oceania), stressing the international character of the Society. The ECS organizes very successful scientific meetings and dedicated workshops that allows a close and fruitful interaction between junior researchers and senior experts in the field. In the last years the ECS organized workshops in Seix (2007 and 2011), Smolenice (2009), Leuven and Logonna-Daoulas (2013), Seillac (2015) and Toulouse (2017) (https://gbiomed.kuleuven.be/apps/lmcs/ecs/workshops/workshops.php). The 8th edition of the ECS workshop entitled “Calcium Signaling in Aging and Neurodegenerative Diseases” (http://8thworkshopecs.com/), which was attended by 62 participants from 11 different countries, was held on 18–20th September 2019, in the beautiful city of Coimbra, Portugal. With over seven centuries, the University of Coimbra in Portugal has a unique tangible and intangible heritage, keystone in the scientific culture of Europe and the World. This University also pursues a policy of constant improvement in various fields, which allow it to reassert the high quality of scientific research and teaching. 

The ECS 2019 workshop scientific program was structured into a plenary lecture, five symposia (two–three presentations each) and two oral communication (five presentations each) and poster sessions (a total of 19), which were dedicated to the most recent advances in understanding calcium signaling in the brain underlying aging and neurodegenerative disorders, as well as technological innovations in calcium imaging. A hands-on session on this topic (calcium imaging) was offered for interested participants on the last day of the meeting. Invited speakers came from a diversity of research institutions, namely from Portugal, Denmark, France, Italy, Poland, Spain, Sweden, United Kingdom (UK) and the United States of America (USA). 

The Scientific Committee integrated ECS board members and researchers from the national and international scientific community and was responsible for the selection of abstracts for oral communication or poster, evaluation of posters in each session and participated as chairs of each symposium when requested. Scientific Committee members also integrated the jury that selected the best oral and poster communications, which were sponsored by the *International Journal of Molecular Sciences* (*IJMS*).

## 2. Plenary Lecture

The plenary lecture was presented by Giles E. Hardingham (Giles E. Hardingham, Edinburgh Medical School, University of Edinburgh, UK), who reviewed past and present research of the group focused on molecular mechanisms that underlie modified calcium signaling pathways leading to degeneration or resilience in neurons and astrocytes. These included the evidence of oxidative stress, mitochondrial dysfunction, inflammation and changes in activity of synaptic *versus* extrasynaptic *N*-methyl-d-aspartate receptors in several neurological disorders. The role of calcium in conveying both degenerative and adaptive protective responses to adverse conditions in the central nervous system through inter- and intracellular signaling pathways was also discussed.

## 3. Symposium 1—“Synaptic Calcium”

This symposium was dedicated to the analysis of changes in synaptic calcium under physiological and pathophysiological conditions in models of neurodegenerative disorders, namely, in Alzheimer’s disease (AD), having contributed three speakers. The first invited communication was presented by Grace Stutzmann (Center for Neurodegenerative Disease and Therapeutics at Rosalind Franklin University, and Department of Neuroscience at Chicago Medical School, Chicago, IL, USA), who described the main features of AD and the consistent lack of effective treatments, which was described to be partly due to an incomplete understanding of disease mechanisms that affect learning and memory, as well as a discrepancy of results between animal models that are used to test therapies and the disease process that occurs in human neurons. By using AD patient-derived neurons (directly transformed and indirectly induced from iPSC-induced pluripotent stem cells) and acute brain slices from 3xTg-AD and APP/PS1 transgenic mice, the authors identified increased release of calcium from the endoplasmic reticulum (ER) occurring through the ryanodine receptors (RyR), and increased expression of isoform RyR2, while compounds that modulate RyRs were able to reverse AD-related pathological changes, including synaptic defects, amyloid and tau aggregation and lysosomal and mitochondrial dysfunction. Data presented was concordant with the hypothesis that aberrant calcium signaling is an early and upstream driver of AD central features, and thus, a potential target for disease-modifying therapeutic strategies.

Ana M. Sebastião (Institute of Pharmacology and Neuroscience at Faculty of Medicine and Institute of Molecular Medicine, University of Lisbon, Portugal) demonstrated how cannabinoids *per se* and both cannabinoids and adenosine, two neuromodulators, interfere with calcium-dependent activity of the tripartite synapse. Endocannabinoids (eCBs) were shown to have a dual effect on hippocampal long-term potentiation (LTP) by inhibiting weak LTP and facilitating strong LTP; in accordance, exogenous activation of cannabinoid receptor 1 (CB1R) inhibited hippocampal LTP, possibly disrupting memory consolidation and affecting brain connectivity relevant for memory processes. Interaction between CB1R and adenosine receptor A2A was demonstrated since A2AR antagonists attenuated the inhibitory role of CB1R agonists on hippocampal LTP, preventing the impairment of memory consolidation. In astrocytes, CB1R activation caused intracellular calcium oscillations and facilitated glutamate uptake, also under the control of A2ARs. These data suggest that the interaction between CB1Rs and A2ARs may be relevant in mitigating cognitive deficits during cannabinoid-based therapies.

Finally, António Garcia (Instituto Teófilo Hernando, and Facultad de Medicina, University Autonoma de Madrid, Spain) reviewed the several evidences demonstrating that central alterations in calcium handling and exocytosis observed in models of neurodegenerative disorders, namely AD, Parkinson’s disease (PD), Huntington’s disease (HD), or amyotrophic lateral sclerosis (ALS), also affect the peripheral sympatho-adrenal axis, as detected in adrenal chromaffin cells (CCs); these changes were hypothesized to occur due to expression of disease-related mutant proteins and/or a secondary adaptation to stress imposed by disease progression. For instance, in a HD R6/1 mouse model, mutant huntingtin was shown to be expressed in CCs, causing decreased exocytotic release along with faster cytosolic calcium clearance; alpha-synuclein, a protein involved in PD neuropathology, was shown to decrease exocytosis in CCs; in ALS SOD1 G93A mice, CCs showed higher exocytosis despite slower release rate; and in AD APP/PS1 mice vesicle secretion was reduced. Changes at peripheral sympatho-adrenal axis may be associated with impairment of fight-or-flight response in patients suffering from these neurodegenerative disorders, potentially limiting their autonomy.

## 4. Symposium 2—“Mitochondrial Calcium”

This session encompassed three oral presentations pointing mostly at the role of mitochondrial calcium homeostasis and its deregulation in age-related pathological conditions. The first talk, by Michael Duchen (University College of London, UK), described how impaired mitochondrial bioenergetic capacity sensitizes neurons to calcium overload, linking mitochondrial dysfunction to neurodegeneration. Using cultured primary neurons from mice knockout for the lysosomal enzyme glucocerebrosidase (GBA), which has emerged as the major genetic risk factor for PD, Michael Duchen presented evidences that mitochondrial dysfunction caused by loss of GBA function renders the neurons vulnerable to a glutamate-induced calcium signal. Indeed, in the gba^−/−^ cells, an irreversible fall in ATP/ADP in response to glutamate, associated with deregulation of calcium homeostasis and mitochondrial depolarization, was observed in contrast to the transient calcium signal and decrease in ATP/ADP ratio caused by glutamate in control cells. Therefore, these relevant findings demonstrate that the lack of bioenergetic capacity to respond to increased energy demand with increased ATP supply renders the neurons vulnerable to otherwise innocuous concentrations of glutamate, which may play a role in neurodegeneration. Strikingly, *gba1^+/−^* neurons showed a behavior that was similar to that of *gba1^−/−^*, consistent with a role of these mechanisms in the pathogenesis of PD.

Autosomal recessive forms of this neurodegenerative disorder are associated with loss-of-function mutations in the *PARK2* and *PINK1* genes, which encode the E3 ubiquitin ligase Parkin and the mitochondrial serine/threonine kinase PINK1 that jointly regulate various mitochondrial quality control mechanisms. Defects in the ER-mitochondrial interface, ER-to-mitochondria calcium transfer, mitophagy, the response to mitochondrial stress, mitochondrial import and the NLRP3 inflammasome pathway were found to occur in primary fibroblasts, neurons and glial cells from Parkin-deficient mice and patients with *PARK2*-linked PD. These interesting results presented by Olga Corti (Institut du Cerveau et de la Moelle épinière, Paris, France) contribute to clarify the role of mitochondrial dysfunction and mitophagy in PD-associated neuronal degeneration and support that PINK1/Parkin-dependent mechanisms play a role beyond autosomal recessive PD.

Maria Ankarcrona (Karolinska Institutet, Stockholm, Sweden) identified TOM70, a subunit of the mitochondrial translocase of the outer membrane (TOM) that is essential for the post-translational import of nuclear-encoded mitochondrial proteins, as a new player regulating pro-survival calcium-transfer at the ER-mitochondria signaling platform. By using different mammalian cell lines, TOM70, but not TOM20, was demonstrated to cluster in distinct OMM foci frequently overlapping with ER-mitochondria contact sites. From the functional point of view, TOM70 depletion specifically was found to impair IP_3_-linked ER to mitochondria calcium transfer due to the capacity of TOM70 to interact with IP_3_-receptors and favor their functional recruitment close to mitochondria. Importantly, the reduced constitutive calcium transfer to mitochondria, dampens mitochondrial respiration, affects cell bioenergetics, induces autophagy and inhibits proliferation in TOM70 depleted cells. Preliminary data revealed that TOM70 protein levels are specifically decreased in human postmortem samples and several AD mouse models suggesting that TOM70 loss-of-function might be involved in the initiation and progression of this age-related neurodegenerative disorder.

## 5. Symposium 3—“ER Calcium”

This session included three oral presentations focused on ER calcium. In the first talk, Jacek Kuznicki (International Institute of Molecular and Cell Biology, Warsaw, Poland) provided evidences supporting that zebrafish and mouse models can help to understand the molecular mechanisms involved in changes of calcium homeostasis that occur in human age-related diseases such as AD, PD HD neurodegenerative pathologies, namely, the mitochondrial calcium uniporter (MCU and the store-operated calcium entry (SOCE)) machinery. Using zebrafish line with *pink1* mutant as a model of PD, Jacek Kuznicki reported that the loss of dopaminergic neurons can be rescued by removal of MCU. The *mcu* knockout line is fertile, has no calcium influx into mitochondria, and is insensitive to MPTP, a known toxin that leads to the loss of dopaminergic neurons in wild type fish. The calcium hypothesis of AD was supported by data obtained by Jacek Kuznicki’s team. In mice with overexpression of either STIM1, STIM2 or Orai1, context recognition was found to decrease only in female Orai1 transgenic mice, but not in males; the epileptic-like phenotype was observed in female mice, suggesting that ORAI1 overexpression may affect neuronal activity in a sex-dependent manner. On the other hand, STIM1 overexpression in neurons in the brain was demonstrated to affect metabotropic glutamate receptor signaling, leading to impairments in long-term depression and alterations in animal behaviour. Jacek Kuznicki also provided interesting findings in mice overexpressing huntingtin mutant with 126 glutamine residues (YAC128), a model of Huntington’s disease (HD). It was found that dysregulation of calcium homeostasis correlates with changes in the gene expression of members of the calciosome, especially increased expression of Hap1, supporting that HAP1A causes the activation of SOCE channels in HD models by affecting IP_3_R1 activity.

Carlos Villalobos (Institute of Biology and Molecular Genetics (IBGM), University of Valladolid and CSIC, Valladolid, Spain) reported that aging potentiates calcium remodeling induced by AD-associated beta amyloid oligomers (Aβo), the most likely neurotoxin involved in AD. In long-term cultures of rat hippocampal neurons that resemble in many aspects aging neurons, it was observed increased resting cytosolic calcium concentration, calcium store content and calcium release from intracellular stores as well as calcium transfer from the ER to mitochondria, together with a decrease in SOCE. At the molecular level, this calcium remodeling was shown associated to changes in the expression of calcium channels including NMDA receptor isoforms, IP_3_ receptor isoforms, the MCU and Orai1/Stim1, the molecular players involved in SOCE. Evidences showing that Aβo exacerbate most of the changes observed in aged neurons leading to enhanced cell death, or to increased susceptibility to cell death induced by excitotoxic and pro-inflammatory insults were also presented. Interestingly, it was shown that Aβo increases ER-mitochondria colocalization and calcium transfer from ER to mitochondria in young neurons in the absence of detrimental effects supporting that Aβo may be a physiological modulator of ER-mitochondria coupling. However, in aged neurons, Aβo suppressed transfer of calcium from ER to mitochondria, decrease mitochondrial potential, enhance generation of reactive oxygen species (ROS) and induce apoptosis.

Paola Pizzo (Department of Biomedical Sciences, University of Padova, and Institute of Neuroscience, CNR, Padova, Italy) presented relevant data supporting that the pathogenicity of PS2 mutants associated with familial AD forms could be linked to depletion of ER calcium content and potentiation of ER-mitochondria juxtaposition affecting key cell functionalities, such as autophagy, mitochondrial activity and dynamics. Indeed, a blocked autophagy, due to an impairment in autophagosome-lysosome fusion, was shown in FAD-PS2 expressing cells. Moreover, in vivo mitochondrial axonal transport results also affected by FAD-PS2 expression. Finally, Paola Pizzo showed evidence demonstrating that mitochondrial respiration and ATP production are reduced by FAD-PS2 expression, suggesting an unbalanced cell metabolism, potentially connected to neurodegeneration.

## 6. Symposium 4—“Calcium in Glia and Stem Cells”

The symposium on “Calcium in Glia and Stem Cells” consisted on two main communications. The first one, given by Martin Lauritzen (Department of Neuroscience, University of Copenhagen, Denmark), addressed the topic “Calcium-dependent mechanisms of cerebral blood flow regulation;” and the second talk was given by Catherine Leclerc (Center for Integrative Biology, Université de Toulouse, Toulouse, France) “Calcium-dependent signalling in glioblastoma stem-like cells.”

The topic of the first lecture dissected the interplay between calcium waves in neurons and the signaling mechanisms triggered in pericytes and smooth vascular cells to control first and second order capillaries dilation and, consequently, blood flow. These mechanisms functionally act as precapillary sphincters, from where dilation propagates upstream to the penetrating arteriole and downstream to higher order capillaries. The research presented took advantage of two-photon microscope calcium imaging in brain blood vessels to study the properties of the blood-brain barrier (BBB) in vivo. Taken together, the results provide insights into the correlation between calcium rises in nerve cells and vascular cells, helping to better understand the mechanisms of vascular control in the brain.

The second lecture discussed important physiological differences of cancer stem cells properties grown at different pH and oxygen conditions. The calcium signaling mechanism and the mitochondrial/ER morphology/function were discussed under the duality of the cancer stem cell niche favoring proliferation or quiescence. By using calcium imaging and RNAseq analysis, the authors investigated proliferation and quiescence of glioblastome stem-like cells (GSLCs) and how they control calcium homeostasis and mitochondrial morphological reshaping associated with GSLCs quiescence, survival and tumor aggressiveness. The authors also provided insights into the calcium-dependent gene expression mediated by potassium channel interacting proteins (KNIPs) signaling and their role in the control of glioblastome prognosis genes and tumor aggressiveness.

## 7. Symposium 5—“Technical Approaches on Calcium Imaging”

The last session was focused on technological approaches on calcium imaging and included two oral communications. Firstly, Leopoldo Petreanu (Champalimaud Center for the Unknown, Lisbon, Portugal) showed the importance of calcium imaging in afferent axons to clarify the organization of cortico-cortical connections in mouse visual cortex. The obtained results showed that long-range cortical connections are organized with tuning-dependent specificity following simple geometrical rules, helping to elucidate the computations implemented by long-range cortical networks. On the second talk, Javier Garcia-Sancho (Institute for Molecular Biology & Genetics (IBGM), University of Valladolid, and Spanish National Research Council, Valladolid, Spain) presented recent advances on genetically encoded calcium indicators (GECIs), targeting subcellular calcium signals in different organelles, and their suitability to perform measurements of calcium dynamics ex vivo and in vivo in transgenic animals. Specifically, novelties related to a new class of GFP fusion protein with aequorin (GAP: GFP-Aequorin-Protein), the first GECI used as calcium probe, were introduced namely some low affinity GAP variants for measuring accurately in high [Ca^2+^] compartments, such as ER or Golgi. The new biosensor was used to measure sarcoplasmic reticulum (SR) calcium dynamics in the muscle of living flies and the changes of the SR calcium content with aging. The results showed that the decrease of muscle activity with aging correlates very well with a parallel decrease of [Ca^2+^]_SR_. due to a disruption of the pump-leak steady state, with a moderate decrease of SERCA expression, no modification of the ryanodine receptors and increase of SR calcium leakiness.

## 8. Oral Communications

The oral communications were divided into two sessions, both held in the second day of the meeting.

Alba Delrio-Lorenzo (University of Valladolid, Valladolid, Spain) presented data supporting the hypothesis that the reduction of the sarcoplasmic reticulum (SR) calcium levels is at the origin of the calcium dyshomeostasis in aging using *Drosophila melanogaster* as a model and a novel quantitative calibrated method to measure SR calcium content, based on a low affinity ratiometric calcium sensor targeted to the SR of thoracic muscles or the ER of brain neurons.

Results obtained in C. elegans worms presented by Javier Alvarez ((University of Valladolid, Valladolid, Spain) showed that submaximal inhibition of SR Ca^2+^ ATPase can extend lifespan suggesting that calcium signaling plays an important role in the aging process.

Using two AD *App* knock-in mouse models at different disease stages, Luana Naia (Karolinska Institutet, Stockholm, Sweden) reported that an initial boost of mitochondrial function may work as a compensatory mechanism and stabilizers of neuronal calcium signaling and mitochondrial function likely have a therapeutic potential in AD and similar neurodegenerative disorders.

P. Gailly (University of Louvain, Brussels, Belgium) reported the activation of an isoform of the transient receptor potential channel (TRPC1), which is highly expressed in the hippocampus, by glutamatergic metabotropic receptors type 5 (mGluR5); to study the mGluR5-TRPC1 pathway, the authors used a TRPC1 knock-out mice, and inhibition of TRPC1 by Pico145 in isolated neurons or brain slices. The mGluR5-TRPC1 pathway was shown to play a role in synaptic plasticity, spatial working memory and memory extinction.

G. Dupont (Université Libre de Bruxelles, Brussels, Belgium) described a computational modeling approach to study the dysregulation of calcium homeostasis that occurs after activation of *N*-methyl-d-aspartate receptors in response to oligomers of beta-amyloid peptide based on experimental data. Calcium influx under these conditions was related with the activation of calcium-activated potassium channels, altering neuronal activity, and disturbances in electrical activity could be explained in the presence of beta-amyloid.

In session 2, Bruno Constantin (University of Poitiers, Poitiers, France) studied how activation of store-operated calcium channels and calcium signaling can regulate growth and self-renewal of neural stem cells derived from the subventricular zone in the adult brain.

M.F. Cano-Abad (Universidad Autónoma de Madrid and Hospital Universitario de la Princesa, in Madrid, Spain) used peripheral blood mononuclear cells from patients with major depressive disorder to evaluate the functionality of purinergic P2X7 ligand-gated receptors and calcium homeostasis, and its correlation with patient’s inflammatory state. The authors showed modified functionality of P2X7 receptors and intracellular calcium deregulation in patient’s cells.

M.K. Oliva (Department of Genetics, University Cambridge, Cambridge, UK) studied the effect of hereditary spastic paralegia (HSP) genes on handling of presynaptic and axonal calcium involving the ER. They developed GAL4-driven genetically-encoded calcium indicator targeted to the lumen of the organelle in motor neurons of wild-type Drosophila and fly mutants for reticulon and REEP genes.

Kushal Kolar (Sars International Centre for Marine Molecular Biology, Bergen, Norway) presented a new calcium imaging analysis platform called ‘Mesmerize’ that can be applied to investigate a wide range of questions related with calcium in different biological systems. Novel analysis tools have been introduced in this platform that allow a quantitative analysis of calcium activity patterns.

Tito Cali (University of Padova, Padova, Italy) presented findings obtained with new-generation sensors able to explore the dynamic nature of organelle contact sites in health and disease. The dynamic nature of the ER-mitochondria interface in the axon of zebrafish sensory neurons and in dendrites and soma of rat primary hippocampal neurons were demonstrated using novel modular genetically encoded sensors engineered to fluoresce when narrow and wide juxtapositions occur between organelles.

## 9. Poster Communications

The poster session consisted of 19 communications that included diverse aspects of the physiology of calcium in thematic areas such as: aging, Alzheimer’s disease; phytochemicals; inflammasome-induced sterile inflammation; cell survival pathways; calmodulin; mitochondria; retina neurodegeneration. Poster presenters came from Germany, Spain, Denmark, Italy, Portugal, UK and Poland.

## 10. Hands-on Activity

The Hands-on on Calcium Imaging was held at the Microscopy Facility of the Center for Neuroscience and Cell Biology (CNC). The 2-hour hands-on activity combined talks and hands-on activities on imaging and image analysis focused on the imaging methodologies applied to study intracellular calcium dynamics. Therefore, participants were introduced to different calcium biosensors, microscopy setups and imaging analysis approaches, taking into consideration the advantages and drawbacks of the different techniques.

## 11. Conclusions

The workshop was very didactic, and all presentations overviewed the progress made in specific topics and covered valuable, recently published data or newly unpublished findings. Some of the novel results have been included in this Special Issue of IJMS. Calcium signaling is essential for many vital functions of the brain and even minor impairments in calcium signaling can lead to deleterious consequences for the nervous system, including neuronal death. Indeed, disturbances in calcium signaling have been implicated in brain aging and in the pathogenesis of various chronic neurodegenerative disorders. The ECS 2019 workshop illustrated the progress in the state of the art in this field, especially in the mechanisms responsible for calcium handling in different cell types and how it is perturbed in aging and age-related brain pathologies. Recent developments in experimental tools for calcium imaging were also reported. A very valuable part of the meeting included stimulating discussions among researchers from different areas of expertise and with young researchers interested in calcium signaling. The free flow of information contributed to intensive interaction between scientists and students, who participated in substantial number in this meeting. This platform is a very good starting point for the next ECS workshop in 2021, which we all are looking forward to.

